# Prevalence of patients with unmet palliative care needs in a University Emergency Department in Germany – a cross-sectional screening study

**DOI:** 10.1186/s12904-025-01892-2

**Published:** 2025-10-07

**Authors:** Kristine Engeleit, Kambiz Afshar, Stephanie Stiel, Nils Schneider, Tanja Schleef, Torben Brod

**Affiliations:** 1 Institute for General Practice and Palliative Care, Hannover Medical School, Carl-Neuberg-Strasse 1, Hannover, 30625 Germany; 2 Department of Emergency Medicine, Hannover Medical School, Carl-Neuberg-Strasse 1, Hannover, 30625 Germany

**Keywords:** Palliative medicine, Palliative care needs, Emergency department, Health services research, P-CaRES

## Abstract

**Background:**

People with life-limiting diseases can benefit from early integration of palliative care, yet early identification of these patients is challenging. Emergency Departments (ED) often serve as the initial contact point for hospital admissions due to acute medical problems, providing an opportunity to recognize palliative care needs. This study aims to determine the prevalence of unmet palliative care needs among patients presenting to the ED of a German university hospital and to assess the proportion of these patients already receiving outpatient palliative care services.

**Methods:**

We conducted a cross-sectional study at the ED of Hannover Medical School over a 30-day period in April and May 2022. All patients aged 18 years or older who were seen by a specialist in either internal medicine or general medicine in the ED were eligible to participate and screened using the Palliative Care and Rapid Emergency Screening Tool (P-CaRES) to identify those with unmet palliative care needs. Data were collected by on-duty physicians. For analysis, we used standard descriptive statistics.

**Results:**

During the study period, 1,122 patients presented to the ED and were seen by a specialist in either internal medicine or general medicine. 1,090 could be included in the study (inclusion rate: 97.1%). At least one life-limiting disease was identified in 280 of the 1,090 patients (25.7%). Of these, 173 patients had two or more potentially unmet palliative care needs, as determined by the P-CaRES screening tool. At the time of assessment, 11.6% of these patients were already receiving outpatient palliative care.

**Conclusion:**

A significant proportion of patients presenting to the ED of a university hospital appear to have unmet palliative care needs according to P-CaRES. However, only a small fraction of these patients were known to have pre-existing outpatient palliative care support which could indicate a considerable gap in palliative care provision. Promoting the integrating of palliative care into emergency medicine could substantially enhance patient care by promoting holistic, patient-centred approaches that extend beyond acute medical treatment and facilitate the early implementation of outpatient palliative care services.

## Background

People with life-limiting diseases can benefit from early integration of palliative care. However, the timely identification of patients with potential palliative care needs is challenging, particularly in the case of non-oncological diseases [1, 2]. Emergency Departments (EDs) serve as critical hubs and are often the entry point for (unplanned) hospital admissions of patients with acute medical problems. However, due to the rapid and acute nature of emergency care, long-term needs of these patients can sometimes be overlooked. ED presentations within the last phase of life are observed on an international scale and are particularly prevalent among individuals diagnosed with dementia, cancer and/or other chronic diseases [3–5]. Consequently, presentation to an ED is considered a critical time point to identify the need for palliative care and to provide palliative care support to patients with life-limiting diseases [4, 6]. The integration of palliative care into emergency medicine is therefore recommended by both emergency medicine and palliative care societies [7].

In Germany, approximately 40% of ED patients are aged 65 years or older, and demographic change will increase this proportion [8]. Older patients are more likely to suffer from chronic, life-limiting diseases that may not be amenable to curative treatment, posing a challenge for emergency physicians [9]. When such patients present acutely to an ED, the physician will often seek curative or life-sustaining measures within the framework of emergency medicine. Yet, this approach does not always meet the individual needs of these patients [10, 11]. Maximal diagnostic and therapeutic measures can be burdensome and may not be in accordance with the patient’s wishes and care preferences, especially in older, multimorbid patients. The goals of palliative care advocate optimal symptom control to improve quality of life, but not prolongation of life at all costs. Early identification of patients with unmet palliative care needs can therefore help to meet the specific needs of patients or their families and improve their quality of life [12, 13]. Thus, presentation to an ED may be a crucial time to identify the potential need for palliative care in patients with chronic, life-limiting diseases.

Several screening tools, including the Surprise Question (SQ) and the Palliative Care and Rapid Emergency Screening Tool (P-CaRES), have been developed to identify these patients in the ED, but have shown wide variation in sensitivity and specificity [14–17]. Implementation in daily practice is often hampered by the high acuity of emergency care and the lack of human resources to administer such screening tools routinely. Estimating the prevalence of patients with unmet palliative care needs in the ED may therefore help to identify the need for increased ED staffing and subsequent palliative care consultation and may also allow targeting of specific subpopulations of patients in the ED to reduce the volume of screening. To date however, only a limited number of studies have estimated the prevalence of patients with palliative care needs at ED presentation. For example, a retrospective study from Portugal found that 78% of ED patients with dementia in their last year of life had unmet palliative care needs. In Austria, Koestenberger et al. reported that 13% of all ED patients screened positive for unmet palliative care needs using the P-CaRES tool [18, 19]. However, no prevalence data are currently available for Germany [12].

Therefore, the aim of this study was to assess the prevalence of patients with potential unmet palliative care needs in an ED of a university hospital in Germany and to estimate how many of these patients already received outpatient palliative care.

## Methods

### Study design

We conducted a cross-sectional study of adult patients presenting to an ED in Germany during a 30-day period in April and May 2022, using the P-CaRES to identify patients with potential unmet palliative care needs [15]. The Ethics Committee of the Hannover Medical School approved the study (03/08/2022; No. 10255_B0_K_2022).

### Setting and recruitment

Patients were recruited from the ED of the university hospital of Hannover Medical School, a tertiary referral centre in northern Germany, serving a diverse patient population. A specialized palliative care service for in-house consultation and a palliative care unit is available on request for all patients in the hospital, including the ED. All patients aged 18 years or older presenting to the ED that were seen by a specialist in either internal medicine or general medicine were considered eligible to participate. Due to the exploratory nature of this screening study and based on clinical experience indicating that a substantial proportion of patients with potential palliative care needs are managed by specialists in general or internal medicine in our ED, we focused on patients under the care of these two specialties. This decision was made to ensure the feasibility of the study within the routine clinical workflows of the ED, whilst allowing for a high inclusion rate through a full census sampling strategy.

### Data collection

Data were collected by the physician on duty either during the initial patient interview or immediately afterwards using a one-page data collection form. All physicians were informed about the purpose and the practical implementation of the study in advance. Sociodemographic data, Emergency Severity Index (ESI) triage category, chief complaint or leading symptom on presentation to the ED, suspected diagnosis at presentation, and patient disposition (admission or discharge) were recorded. The chief complaint was coded according to the Canadian Emergency Department Information System (CEDIS), using the German translation by Brammen et al. [20]. The suspected diagnosis was coded according to the International Statistical Classification of Diseases and Related Health Problems, 10th Revision, German Modification (ICD-10-GM) without subgroups. This is the official classification for coding diagnoses in outpatient and inpatient care in Germany [21].

To identify unmet palliative care needs, the P-CaRES, developed and validated in the United States for patients in ED, was used [15, 16]. The P-CaRES tool was previously translated into German by Koestenberger et al. in Austria [19]. Prior to its use in the present study, only minor changes were made to this German version. First, underlining was reintroduced into the questionnaire, which had been included in the original American version but omitted by the Austrian authors. Second, the translation of the term “goals of care” in the second section of P-CaRES was adapted to reflect a broader interpretation that is more in line with the use of the term in the German health care context than the formulation chosen in the Austrian version. These adaptations were made by an interdisciplinary and multi-professional team with expertise in palliative medicine, general medicine, emergency medicine and health sciences. P-CaRES consists of two parts with a total of 13 items. The first part assesses whether the patient has one of eight life-limiting conditions, such as advanced oncological disease or advanced heart failure. Multiple answers are possible. If no item is checked, the screening is completed with no unmet palliative care need. If at least one life-limiting disease is identified, the screening continues. The second part checks whether the patient has unmet palliative care needs. Survey items in the second part include frequent consultations, defined as two or more visits to the ED or hospital admissions in the last six months, uncontrolled symptoms, functional decline, uncertainty about goals of care and/or caregiver burden. A third-party history with the caregiver has to be taken if necessary to clarify an increasing care problem due to overburdening of relatives. The surprise question (“You would not be surprised if this patient died within 12 months?“) is incorporated as the final item in the P-CaRES screening tool, thus concluding the second part of the screening. If at least two of five items are checked in part two, the patient has been positively screened for unmet palliative care needs. For this screening-positive group of patients, previous palliative care interventions and involvement of integrated service providers were assessed by the physician on duty during completion of the data collection form in direct consultation with the patient.

### Data analysis

Data collection was completely anonymous. Statistical analyses were conducted using IBM SPSS Statistics version 27 (IBM Corporation, Armonk, NY, USA). Absolute (n) and relative frequencies (%) were calculated for categorical variables, and means with standard deviation were calculated for interval scaled variables.

## Results

**Fig. 1:**
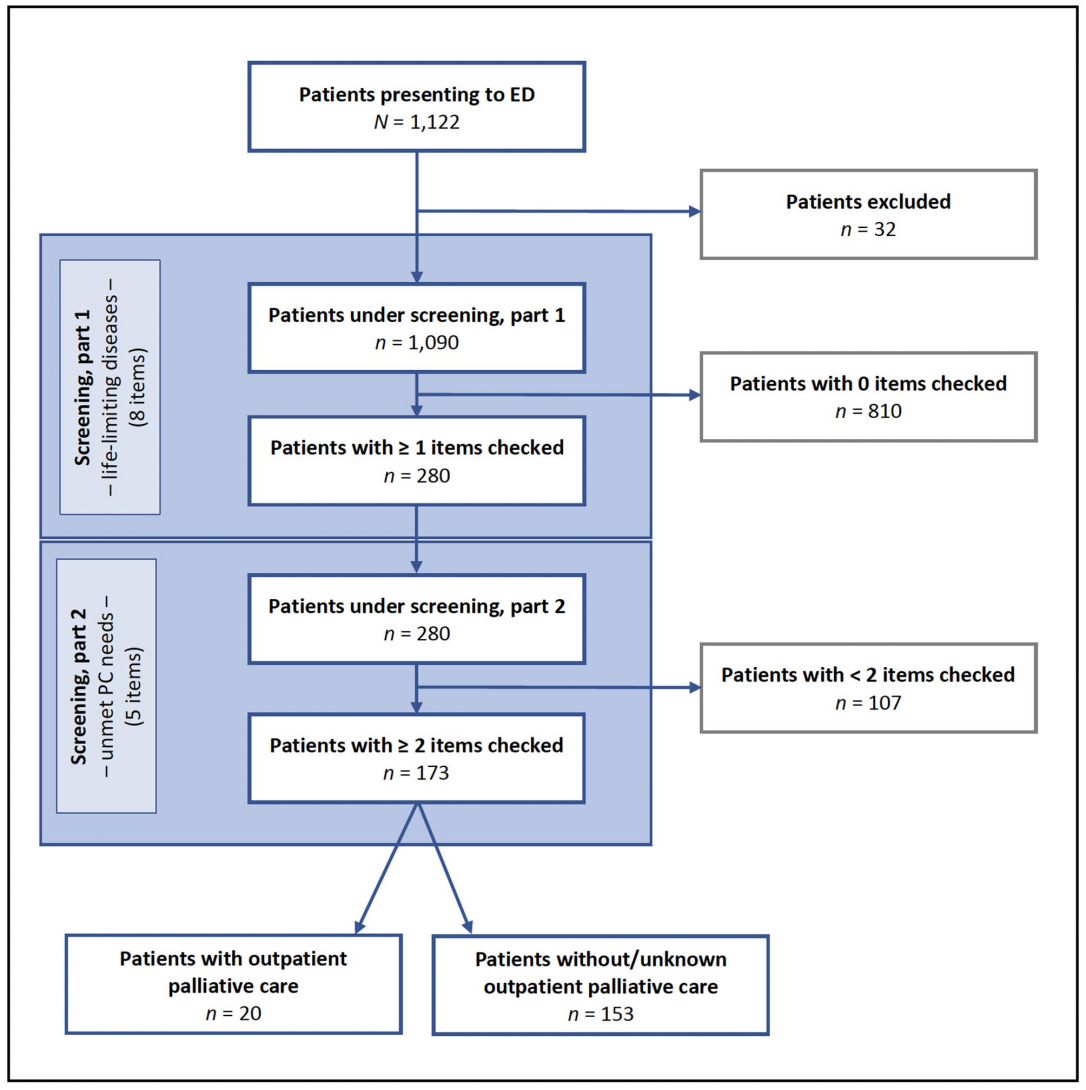
Study flow chart and screening process using the P-CaRES. PC Palliative care/P-CaRES Palliative care and rapid emergency screening tool

During the four-week study period, 1,122 patients presented to the ED and were seen by a specialist in either internal or general medicine, thereby fulfilling the basic eligibility criteria. Of these, 1,090 patients were included in the study, while 32 were excluded due to the severity of illness (e.g. cardiac arrest), resulting in an inclusion rate of 97.1% (Fig. 1).

The mean patient age was 56.2 years, and 50.1% of the patients were male. Most patients were triaged as ESI 3 or 4 and could be discharged home after treatment in the ED (56.7%). Table 1 summarises the patient characteristics.

**Table 1:** Characteristics of all patients presenting during the study period, ESI triage, and whereabouts after presentation

Variable		
Age [years] (*N* = 1,086)	56.2	± 20.8
Age subgroup [years] (*N* = 1,086)		
≤ 19	25	(2.3%)
20–29	137	(12.6%)
30–39	123	(11.3%)
40–49	125	(11.5%)
50–59	159	(14.6%)
60–69	182	(16.8%)
70–79	150	(13.8%)
80–89	159	(14.6%)
≥ 90	26	(2.4%)
Sex (*N* = 1,081)		
Male	542	(50.1%)
Female	539	(49.9%)
ESI (Emergency Severity Index) level (*N* = 1,090)		
Not specified	45	(4.1%)
1 (life-saving interventions needed)	11	(1.0%)
2 (high risk situation at hand)	123	(11.3%)
3 (multiple resources for stabilization needed)	577	(52.9%)
4 (one resource for stabilization needed)	267	(24.5%)
5 (no resources necessary)	67	(6.1%)
Whereabouts after presentation (*N* = 1,090)		
Not specified	8	(0.7%)
Outpatient setting	618	(56.7%)
Inpatient MHH	376	(34.5%)
Inpatient external hospital	80	(7.3%)
Death	3	(0.3%)
Outpatient setting (against medical advice)	5	(0.5%)

Data are presented as mean ± standard deviation or *n* (%). Values may not add to 100% due to rounding

The most common chief complaints prompting ED presentation were shortness of breath (13.4%), abdominal pain (13.3%), chest pain (9.4%), weakness/worsening of general condition (6.3%), and fever (6.1%). The most common suspected diagnoses according to ICD chapter classification were diseases of the circulatory system (19.4%), diseases of the digestive system (13.6%), infectious and parasitic diseases (8.6%), diseases of the respiratory system (8.5%), and symptoms due to abnormal laboratory findings (8.2%).

### P-CaRES screening tool

In the first part of the P-CaRES screening, at least one life-shortening disease was identified in 280 of the 1,090 patients (25.7%) who presented to the ED. Of these patients, 243 had one, 35 had two, and 2 had three life-shortening diseases. The three most common diseases were: oncological diseases (42.9%), advanced heart failure (23.9%) and dementia/neurological disease (16.8%) (Table 2).

**Table 2:** P-CaRES, part 1 – distribution of life-shortening diseases (*N* = 280)

Item			
1.1 Advanced dementia or central nervous system diseases	47	(16.8%)	
1.2 Advanced oncological disease	120	(42.9%)	
1.3 End stage renal disease	24	(8.6%)	
1.4 Advanced chronic obstructive pulmonary disease	14	(5.0%)	
1.5 Advanced heart failure	67	(23.9%)	
1.6 End stage liver disease	26	(9.3%)	
1.7 Septic shock	4	(1.4%)	
1.8 Provider discretion – high chance of accelerated death	17	(6.1%)	

Data are presented as *n* (%)

In the second part of the P-CaRES questionnaire, 173 of the 280 patients with at least one life-shortening disease had two or more potentially unmet palliative care needs. The most common checked items were the surprise question (61.1%), followed by at least two ED visits or hospital admissions in the previous 6 months (44.6%) (Table 3).

**Table 3:** P-CaRES, part 2 – frequency of unmet palliative care needs (*N* = 280)

Item			
2.1 Frequent ED visits or hospital admissions	125	(44.6%)	
2.2 Uncontrolled symptoms	71	(25.4%)	
2.3 Functional decline	77	(27.5%)	
2.4 Uncertainty about goals of care and/or caregiver distress	25	(8.9%)	
2.5 Surprise question	171	(61.1%)	

Data are presented as *n* (%)

Based on all 1,090 patients presenting to the ED during the study period, the prevalence of patients with unmet palliative care needs in this patient population was 15.9%.

The mean age of patients with a positive P-CaRES screening in this study was 70.0 years, and 60.6% were male. The most common chief complaint was shortness of breath (26.0%), followed by generalised weakness (15.0%) and abdominal pain (7.5%). Life-limiting diseases of the circulatory system (19.7%) predominated, followed by oncological diseases (17.3%). Overall, 49.1% of the 173 patients suffered from an oncological disease and 78.8% had to be admitted to an inpatient ward (Table 4).

**Table 4:** Patient characteristics, most common leading symptoms, and main diagnosis of positive screened patients (*N* = 173)

Variable		
Age [years] (*N* = 172)	70.0	± 15.4
Age subgroup [years] (*N* = 172)		
≥ 60	128	(74.5%)
Sex (*N* = 170)		
Male	103	(60.6%)
Female	67	(39.4%)
Most common leading symptoms (*N* = 173)		
Shortness of breath	45	(26.0%)
General weakness	26	(15.0%)
Abdominal pain	13	(7.5%)
Change of consciousness	12	(6.9%)
Abnormal laboratory values	8	(4.6%)
Most frequent main diagnosis according to ICD classification (*N* = 173)		
Diseases of the circulatory system	34	(19.7%)
Oncological diseases	30	(17.3%)
Diseases of the digestive system	23	(13.3%)
Respiratory diseases	20	(11.6%)
Infectious diseases	15	(8.7%)
Underlying life-limiting condition (*N* = 173)		
Oncological	85	(49.1%)
Non-oncological	88	(50.9%)
Whereabouts after presentation (*N* = 170)		
Outpatient	36	(21.2%)
Inpatient	135	(78.8%)

Data are presented as mean ± standard deviation or *n* (%)

Only 20 patients (11.6%) who screened positive for unmet palliative care needs were already receiving outpatient palliative care. Another 28 patients (16.2%) were unsure or unable to report whether they were receiving such services, and for one patient no information was documented on the data collection form.

## Discussion

In this cross-sectional study of patients, presenting to the ED of a university hospital in Germany, we found that approximately 15.9% of all adult patients who were seen by a specialist in either internal medicine or general medicine had potential unmet palliative care needs according to the P-CaRES questionnaire. Only 11.6% of these patients were known to have pre-existing support from an outpatient palliative care service.

### Identification of patients with unmet palliative care needs

Koestenberger et al. reported similar findings from a non-university tertiary care medical centre in Austria in unselected patients, showing that 13.2% of patients presenting to an ED had an unmet palliative care need [19]. Similar to our study, most of these patients required admission to an inpatient ward and their main complaints were dyspnoea and general weakness. For inpatients, Meffert et al. showed that in a sample of 39,849 patients, 6.9% had palliative care needs, but only 2% of them received palliative care treatment while in hospital [22]. In all three studies, a significant percentage of patients needed palliative care support in both the ED and hospital wards, supporting mandatory screening in both settings to identify patients in need.

Only 11.6% of the positively screened patients with unmet palliative care needs already received outpatient palliative care services prior to this study and only 2% received palliative care treatment as inpatients in the study by Meffert et al. [22]. Although data collection in the study from Meffert et al. took place in 2004/2005 and substantial efforts have been made since then to establish dedicated palliative care units and in-house consultation services in many hospitals in Germany, our findings still indicate lack of awareness for palliative care needs.

The outpatient setting is where the identification of potential palliative care needs usually takes place, with general practitioners playing an important role [23, 24]. EDs can make an additional contribution raising awareness of palliative care for patients with chronic, life-limiting diseases in the event of unplanned hospital admission or sudden, crisis-related deterioration in health, who have not yet been considered for palliative care at the time of presentation in the ED. EDs could then either provide advice and make arrangements with social services as part of care planning for those patients who remain in hospital or, for those who are discharged, include a recommendation for outpatient palliative care support in the discharge report.

### Provision of palliative care in the ED setting

As shown by both Koestenberger et al. and this study, uncontrolled symptoms like shortness of breath or generalised weakness account for a significant number of ED visits and subsequent hospital admissions [19]. Early initiation of palliative care support appears to be crucial to counteract these findings and provide patients with the necessary support system. Dedicated palliative care nurses or, if not available in the ED, specially trained emergency nurses or physician assistants could fill this gap, screening patients and providing initial palliative care counselling early during treatment, thereby contributing to a reduction in the length of hospital stay [25]. Given the current shortage of available hospital beds and increasing reports of ED overcrowding in Germany, it seems crucial to initiate outpatient palliative care early to reduce the number of ED visits and subsequent hospital admissions, as shown by both Wang and Seow et al. [26–28]. Even when specialized outpatient palliative care is available, some medical emergencies cannot be managed in this setting and by advance care planning, requiring presentation to the ED [29, 30]. Emergency medicine societies in Germany have therefore called for the introduction of a structured interdisciplinary and multi-professional treatment team, consisting of experienced acute and palliative care physicians, as a decision-making aid in emergency medicine, in order to formulate early therapeutic goals in accordance with the patient’s wishes [7].

### Methodological reflection

In addition to uncontrolled symptoms and frequent consultations with healthcare providers, a positive response to the surprise question was a key factor for positive P-CaRES screening in this study. As shown by Haydar et al., the modified surprise question is a simple trigger for the need for palliative care, identifying those at higher risk of in-hospital mortality and resource use in the ED [31]. In heart failure patients in the ED, Aaronson et al. showed that the surprise question can help identify patients who would benefit from early palliative care involvement [32]. In this study, the surprise question was answered ‘yes’ by the emergency physician in 98.8% of patients with positive P-CaRES screening, considerably higher than the 53.1% described by Koestenberger et al. [19]. The inconsistency of the study results and the obvious pre-selection by the first screening part of our study raise the question of whether reliance on this screening tool alone is sufficient to identify patients with unmet palliative care needs in the ED. Additionally, and in contrast to other screening tools for identifying patients with potential palliative care needs, the surprise question focusses on the end of life. Different studies that evaluated the use of the surprise question could demonstrate, that a high proportion of patients, approximately about 40%, for whom the question could be answered with “yes”, died within a period of a few months [32]. This is not fully consistent with the concept of integrating palliative care at an early stage in the disease trajectory [33, 34]. Therefore, it is crucial that a positive screening result is followed by a comprehensive needs assessment to provide patient-centred and needs-oriented palliative care measures.

### Limitations

Our study has several limitations. First, the study was conducted in the ED of a university hospital with a high amount of complex and multimorbid patients, not comparable to smaller non-university EDs. Secondly, only patients treated in the ED by either an internal medicine specialist or a general practitioner were included due to explorative nature of this study and the practical experience that a large proportion of potential palliative care patients are seen by these two specialities. This approach reduced the sample size and excluded patients with trauma or neurological emergencies. Third, the physician on duty completed the form either during the initial patient interview or immediately afterwards, which introduced the possibility of variability in the depth of assessment due to workload and time constraints. Also, it is unclear whether all patients, who screened positive in the P-CaRES exactly knew if they were already receiving outpatient palliative care (recall bias), possibly underestimating the amount of screening for palliative care needs that has already taken place in the outpatient setting.

## Conclusion

This study provides important insights into the prevalence of unmet palliative care needs in patients presenting to a university ED in Germany. Fostering the integration of palliative care in emergency medicine could lead to a major improvement in the care of patients with life-limiting diseases by promoting holistic, patient-centred care that goes beyond acute medical treatment. As it is estimated that by 2040, the number of people with palliative care needs will increase by up to 25.0% it seems crucial to develop optimal strategies for the implementation and delivery of palliative care in this fast-paced environment [35].

## Data Availability

The datasets used and/or analysed during the current study are available from the corresponding author on reasonable request.

## Abbreviations

ED: Emergency department
P-CaRES: Palliative care and rapid emergency screening tool
SQ: Surprise Question
ESI: Emergency severity index
CEDIS: Canadian emergency department information system
